# A Case of Idiopathic Hypereosinophilic Syndrome With High WT1 (Wilms' Tumor Gene) mRNA

**DOI:** 10.7759/cureus.116864

**Published:** 2026-09-24

**Authors:** Keiichi Tamai, Yuzo Suzuki, Teie Egawa, Hiroki Kikuchi, Shiho Uchiyama, Ryosuke Kikuchi, Hiroshi Yokoyama

**Affiliations:** 1 Gastroenterology, JR Sendai Hospital, Sendai, JPN; 2 Internal Medicine, JR Sendai Hospital, Sendai, JPN; 3 Dermatology, JR Sendai Hospital, Sendai, JPN

**Keywords:** corticosteroid, erythema multiforme, hypereosinophilia, idiopathic hypereosinophilic syndrome, wt1

## Abstract

A 59-year-old man presented with fever, erythema multiforme, and pancreatic swelling. Laboratory tests revealed severe peripheral hypereosinophilia without a history of relevant medication or evidence of infection. Bone marrow analysis revealed no blast proliferation or leukemia-associated fusion genes. Notably, Wilms' tumor gene (WT1) mRNA was markedly elevated at 26,000 copies/µg RNA. Idiopathic hypereosinophilic syndrome was diagnosed, and oral corticosteroid therapy was initiated, resulting in rapid and complete improvement. This case highlights a rare presentation of idiopathic hypereosinophilic syndrome with high WT1 mRNA, which helps in the differential diagnosis between secondary (reactive) and idiopathic hypereosinophilic syndrome (HES).

## Introduction

Hypereosinophilic syndrome comprises a group of disorders characterized by increased eosinophils in the peripheral blood and end-organ damage due to eosinophilia [1]. There are several causes such as allergies, infections, drugs, and monoclonal proliferation of eosinophils as hematological malignancies. When no underlying etiology is identified, the patient is diagnosed with idiopathic hypereosinophilic syndrome.

The Wilms' tumor gene (WT1) is expressed in the developing fetal kidney, gonads, and in Wilms' tumors. Notably, WT1 is overexpressed in acute myeloid leukemia but is rarely detected in healthy bone marrow or peripheral blood mononuclear cells [2]. Thus, WT1 mRNA is widely recognized as a tumor-associated marker. Herein, we report a rare case of idiopathic hypereosinophilic syndrome that presented with remarkably high levels of WT1 mRNA, despite the absence of any identifiable underlying causes, including driver gene rearrangements.

## Case presentation

A 59-year-old man was admitted to our hospital with fever and skin erythema. Seven weeks prior to admission, he developed a fever, and six weeks before admission, he was evaluated at a medical clinic and diagnosed with an acute upper respiratory tract infection. He was treated with clarithromycin, dextromethorphan, bromhexine, and a combination cold medication containing salicylamide, acetaminophen, anhydrous caffeine, and promethazine methylenesuccinate for five days. Subsequently, the patient noticed erythema on his extremities and inguinal regions. Twenty-four days before admission, he became febrile again and visited an emergency center. A throat swab for rapid antigen testing was negative for group A streptococcus, adenovirus, severe acute respiratory syndrome coronavirus 2 (SARS-CoV-2), and influenza A and B. At a local dermatology clinic, he was suspected of having erythema multiforme secondary to infection. Treatment with amoxicillin for seven days and betamethasone/delgocitinib ointment was started, but the erythema did not improve. Three days before admission, he was evaluated at another medical clinic. A rapid test for *Mycoplasma pneumoniae* was negative. The patient was referred to our hospital and admitted on the same day.

At presentation (day 0), the patient had itching and headache, but no abdominal pain. The patient had been taking sitagliptin, ipragliflozin L-proline, metformin, imeglimin, and pitavastatin for at least two years. On examination, his body temperature was 37.2°C, blood pressure was 109/71 mmHg, his heart rate was 102 beats per minute, and oxygen saturation was 97%, while he was breathing ambient air. His body weight was 76 kg, and height was 178 cm. Laboratory tests are shown in Table 1, showing marked eosinophils (48.2%, 11,038/µL). myeloperoxidase-anti-neutrophil cytoplasmic antibody (MPO-ANCA) and proteinase 3-anti-neutrophil cytoplasmic antibody (PR3-ANCA) are negative. He had a history of colonic diverticulitis 10 years ago. He had no known adverse reactions to medications. He was a lifelong nonsmoker and a social drinker.

**Table 1:** Laboratory findings on admission. NA: not assessed.

Test name	Value	Unit	Reference range
White blood cells	22,900	/μL	3,300-8,600
Red blood cells	425	10^4/μL	435-555
Hemoglobin	13.1	g/dL	13.7-16.8
Platelets	356,000	/μL	158,000-348,000
Neutrophils	46.7	%	39.7-70.6
Lymphocytes	3.8	%	23.1-49.9
Monocytes	1	%	4.4-10.0
Eosinophils	48.2	%	0.8-5.4
Basophils	0.3	%	0.2-1.4
Prothrombin time - International normalized ratio	0.97	NA	0.85-1.15
Activated partial thromboplastin time	27.9	sec	24-34
Total bilirubin	0.6	mg/dL	0.4-1.5
Gamma-glutamyl transferase	281	U/L	13-64
Aspartate aminotransferase	14	U/L	13-30
Alanine aminotransferase	28	U/L	10-42
Alkaline phosphatase	473	U/L	38-113
Lactate dehydrogenase	341	U/L	124-222
Cholinesterase	170	U/L	240-486
Na	140	mmol/L	138-145
K	4.3	mmol/L	3.6-4.8
Cl	104	mmol/L	101-108
Ca	8.7	mg/dL	8.8-10.1
Blood urea nitrogen	15.2	mg/dL	8.0-20.0
Creatinine	1.04	mg/dL	0.65-1.07
Uric acid	4.9	mg/dL	3.7-7.8
Creatine kinase	59	U/L	59-248
Albumin	2.2	g/dL	4.1-5.1
Total protein	5.8	g/dL	6.6-8.1
Amylase	32	U/L	44-132
C-reactive protein	11.22	mg/dL	0.00-0.14
Immunoglobulin G	1,187	mg/dL	861-1,747
Immunoglobulin A	302	mg/dL	93-393
Immunoglobulin M	63	mg/dL	33-183
Rapid plasma reagin	-	NA	-
Treponema pallidum latex agglutination	-	NA	-
Hepatitis B surface antigen	-	NA	-
Hepatitis C virus antibody	-	NA	-
N-terminal pro-B-type natriuretic peptide	604	pg/mL	0-125
Antinuclear antibody	< x40	NA	< x40
Soluble interleukin-2 receptor	9,627	U/mL	122-496
Proteinase 3 - Anti-neutrophil cytoplasmic antibody	-	NA	-
Myeloperoxidase - Anti-neutrophil cytoplasmic antibody	-	NA	-
Vitamin B12 (day 4)	353	pg/mL	180-914
Folic acid (day 4)	7.4	ng/mL	>4

On day 0, erythema was present on his extremities, inguinal region, abdomen, and back (Figures 1A, 1B). Chest and abdominal radiography showed no remarkable findings (data not shown). Contrast-enhanced CT showed an enlarged pancreatic tail with poor enhancement, raising the possibility of acute pancreatitis (Figure 1C). An electrocardiogram demonstrated normal sinus rhythm with a heart rate of 96 beats/min (data not shown). Ultrasonography demonstrated normal cardiac wall motion and no pericardial effusion (data not shown). On day 1, esophagogastroduodenoscopy revealed several erosions in the gastric antrum (data not shown). Multiple biopsies were performed, which showed no specific inflammation. On day 2, total colonoscopy revealed no remarkable findings (data not shown).

**Figure 1:**
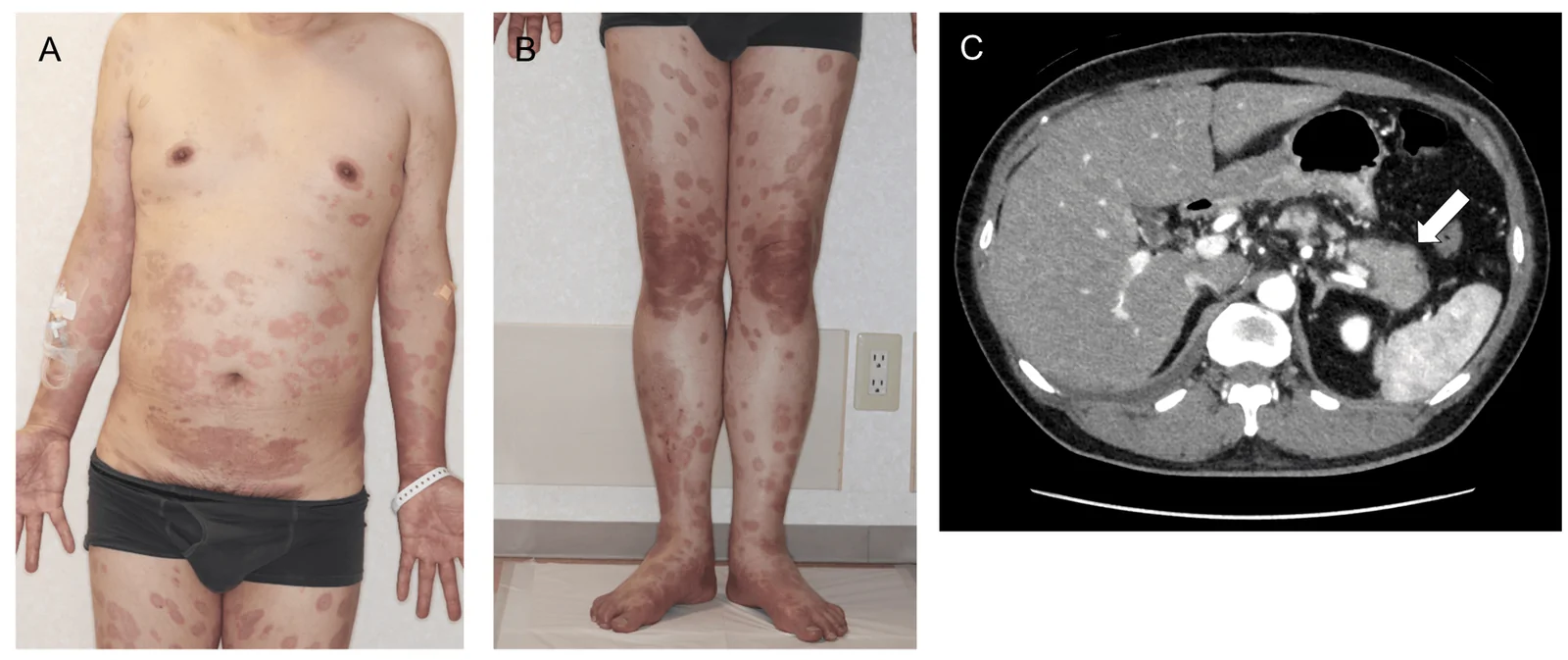
Cutaneous and pancreatic lesions. (A) and (B) Erythema multiforme on admission. (C) A swelling of pancreatic tail (arrow) by contrast-enhanced CT.

Because the erythema multiforme was widespread, oral prednisolone was initiated at 25 mg on day 0 in the evening, and 50 mg of prednisolone was continued on days 1 and 2. The dose of prednisolone was gradually tapered and discontinued on day 16. The erythema multiforme subsequently improved, and eosinophils were decreased (Table 2).

**Table 2:** Time course of eosinophil counts.

Days	Reference range	0	1	4	8	15	23	30
White blood cells (/µL)	3,300-8,600	22,900	27,700	17,400	9,100	6,100	6,400	5,200
Eosinophils (/µL)	70-440	11,038	13,158	6,490	419	421	794	489
Eosinophils (%)	0.8-5.4	48.2	47.5	37.3	4.6	6.9	12.4	9.4

The bone marrow aspirate obtained on day 2 revealed a normocellular marrow. The myeloid lineage showed a mild increase, predominantly driven by marked eosinophilia, with eosinophils accounting for 52.0% of all nucleated cells (Figures 2A, 2B). Eosinophilic maturation was sequential, and the majority of these cells were morphologically mature, with mature, segmented eosinophils comprising 30.4% of the total nucleated cells. In contrast, the erythroid lineage was decreased, resulting in an elevated myeloid-to-erythroid (M:E) ratio of 8.69. Megakaryocytes were normal in number and morphology. Blasts were not increased, accounting for only 0.6% of total nucleated cells. By flow cytometry, there is no specific population.

Cytogenetic and molecular analyses were performed to evaluate the clonal nature of the eosinophilia. Chromosomal analysis of the bone marrow cells by G-banding showed a normal male karyotype of 46, XY in all examined metaphases. Molecular screening was negative for the FIP1L1::PDGFRα fusion gene. Major hematological fusion transcripts, including BCR-ABL1 (major, minor, and micro break-point cluster regions) and CBFB::MYH11, were not detected. Transcripts including PML::RUNX1, ETV6::RUNX1, TCF3::PBX1, KMT2A::AFF1, and KMT2A::MLL3 were also negative. WT1 mRNA showed 26,000 copies/µgRNA (upper limit of normal: < 50). FIP1L1::PDGFRα was negative by fluorescence in situ hybridization. A skin biopsy of the erythema multiforme on day 2 showed that a mild edematous dermis and eosinophils were present intravascularly (Figures 2C, 2D).

**Figure 2:**
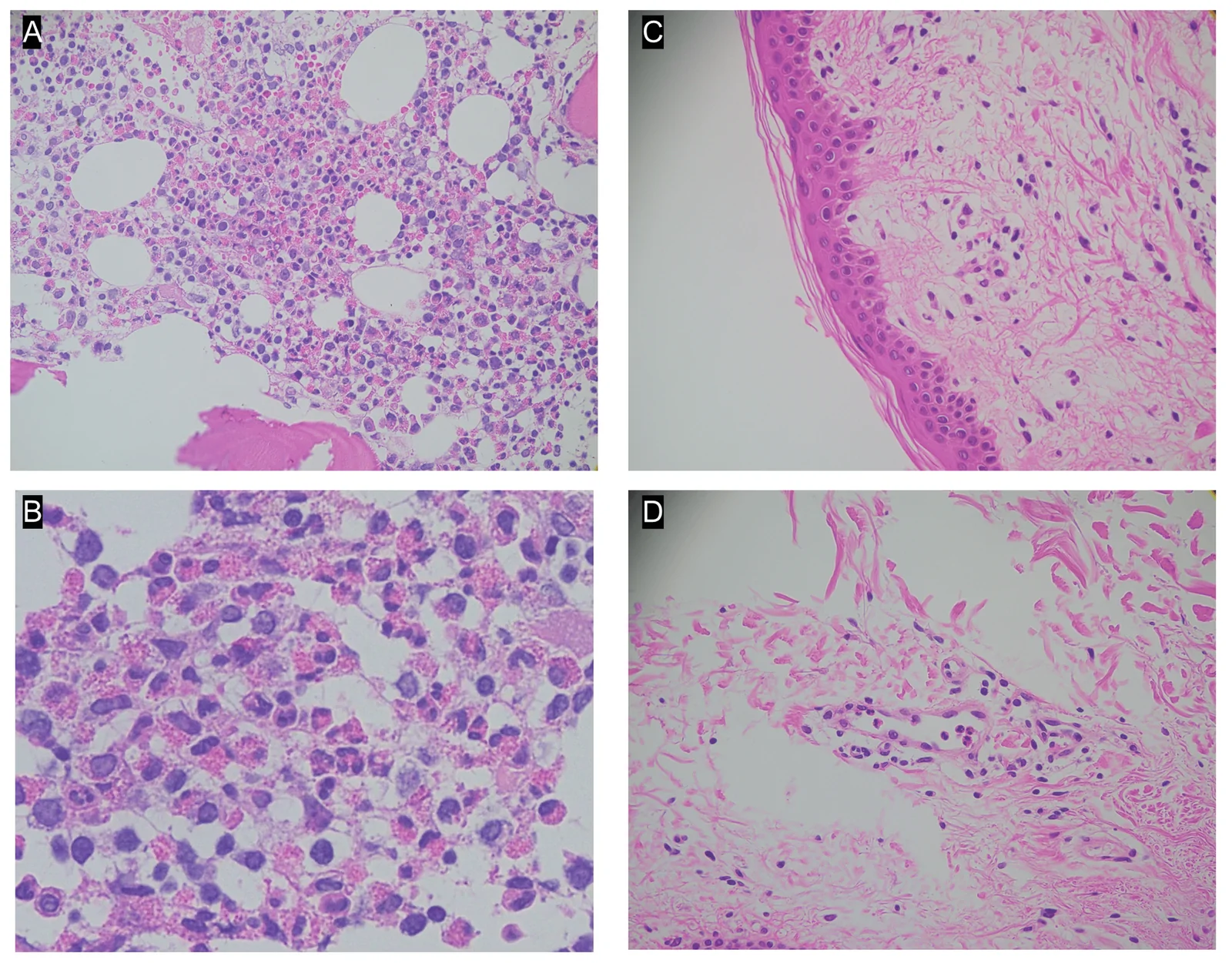
Pathological findings. (A) and (B) Bone marrow biopsy. Hematoxylin-eosin (H&E) staining shows hypercellular marrow with marked eosinophilia. (A) x40, (B) x60. (C) and (D) Skin biopsy stained with hematoxylin-eosin. A mild edematous dermis and eosinophils were present intravascularly, x40.

The patient was discharged on day 5. By day 30, the erythema multiforme had completely resolved, and the eosinophil count was almost within the normal range (Table 2). On day 135, a contrast-enhanced CT revealed no abnormal findings in the pancreas.

## Discussion

Eosinophils are derived from myeloid progenitors in the bone marrow mainly through the action of three hematopoietic cytokines: IL-3, IL-5, and granulocyte-macrophage colony-stimulating factor (GM-CSF) [3]. Eosinophilia is observed in patients with various inflammatory and allergic conditions, as well as diverse hematologic malignancies [4]. Peripheral blood eosinophilia can be divided into mild (0.5-1.5 x 10^9^/L), moderate (1.5-5 x 10^9^/L), and severe (> 5 x 10^9^/L) [5]. Hypereosinophilia is defined as a state where marked and persistent eosinophilia has been documented or marked tissue eosinophilia is observed [5]. Hypereosinophilic syndrome is defined as the presence of hypereosinophilia accompanied by organ damage and/or dysfunction. Idiopathic hypereosinophilic syndrome is classified by the absence of an underlying cause of hypereosinophilia or evidence of a reactive or neoplastic condition/disorder along with end-organ damage attributable to hypereosinophilia [5]. In contrast, reactive hypereosinophilia is characterized by having an underlying inflammatory, neoplastic, or other disease or condition known to produce hypereosinophilia. Major causes include infection (mostly helminthic), drugs (anticonvulsants, antibiotics, sulfa drugs, antirheumatics, allopurinol), pulmonary eosinophilia, and autoimmune diseases such as polyarthritis [6]. Given the diverse etiologies of reactive hypereosinophilia, comprehensively excluding all potential causes remains challenging. In the present case, the patient presented with no specific symptoms except for pruritus associated with erythema. Computed tomography, esophagogastroduodenoscopy, and total colonoscopy revealed unremarkable findings, rendering infection unlikely. There was no history of relevant drug exposure. MPO-ANCA and PR3-ANCA are negative, suggesting that autoimmune diseases such as polyarthritis were unlikely. Based on these clinical and laboratory findings, reactive hypereosinophilic syndrome could be excluded.

The Wilms' tumor gene codes for a zinc-finger transcription factor expressed in few normal adult tissues including bone marrow and peripheral blood cells [7]. By contrast, it is overexpressed in many types of hematopoietic disorders including acute and chronic leukemias and myeloproliferative disorders [8]. Recently, WT1 levels have been recognized as a minimal residual disease marker and useful for prediction of outcome in acute myeloid leukemia [9]. Overall, WT1 is strongly related to the hematological malignancy, not benign diseases. Meanwhile, Cilloni et al. reported that the amount of WT1 transcripts discriminates reactive eosinophilia from idiopathic hypereosinophilic syndrome or chronic eosinophilic leukemia [10]. Bone marrow samples from normal donors expressed a mean value of WT1 copies of 32±19 (WT1 copies/10,000 ABL copies). In case of reactive hypereosinophilic syndrome, WT1 copies are 31.5±18, whereas chronic eosinophilic leukemia patients and hypereosinophilic syndrome patients showed 611±667 and 633±333, respectively. According to this study, WT1 mRNA was 26,000 copies/µgRNA in the present case, suggesting that reactive hypereosinophilic syndrome is highly unlikely. On the other hand, Legrand et al. reported that there was no difference in WT1 copy numbers between patients with FIP1L1::PDGFRA-positive disease and those with secondary hypereosinophilic syndrome [11]. The upstream regulation of WT1 in eosinophils is still largely unknown. GATA is essential for the WT1 gene enhancer in acute leukemia and solid tumor cell lines [12]. Further studies will be required to elucidate the role of WT1 in eosinophilia.

Clinical manifestations of hypereosinophilic syndrome are markedly heterogeneous [6]. The involved organs include the skin, the heart, the nervous system, the lung, the gastrointestinal system, the hematopoietic system, and the kidneys. In patients with idiopathic hypereosinophilic syndrome (HES), skin manifestations (such as rash in 7.9%, pruritus in 5.3%, and edema in 6.6%) have been reported at presentation [13]. Eosinophilic pancreatitis is a rare disorder [14]. The incidence of an increased number of eosinophils is <1% in all pancreatic specimens [15]. Most eosinophilic pancreatitis cases present as a diffuse enlarged head or tail of the pancreas, which is not consistent to this case with local swelling at pancreatic tail. In this case, although we could not identify the cause of skin lesion and pancreatic swelling pathologically, these injuries were likely associated with hypereosinophilia because of the chronological relationship.

Several treatment options are available, including corticosteroids, hydroxyurea, interferon-alpha, and immunomodulatory agents [16]. Corticosteroids are the most widely used and effective therapeutic agents. Eighty-five percent of patients on steroid monotherapy achieved a partial or complete remission within one month of therapy [17]. The response to corticosteroid therapy is generally rapid, with reports of eosinophilia resolving within hours [16]. In this case, the response to corticosteroid was good, and the absence of eosinophils in the extravascular lesion of the skin was likely due to the specimen being obtained on day 2 (after starting treatment).

## Conclusions

We report a rare case of idiopathic hypereosinophilic syndrome presenting with erythema multiforme, characterized by markedly elevated WT1 mRNA levels despite the absence of any identifiable driver gene abnormality or reactive cause. This finding supports previous reports that WT1 transcript levels can help distinguish idiopathic hypereosinophilic syndrome or chronic eosinophilic leukemia from reactive eosinophilia, and suggests that WT1 mRNA quantification may serve as a useful adjunct in the diagnostic work-up of hypereosinophilia. The patient responded promptly and completely to corticosteroid therapy, consistent with the generally favorable short-term response reported for idiopathic hypereosinophilic syndrome. Further accumulation of similar cases and investigation into the mechanisms driving WT1 overexpression in eosinophilic disorders are warranted to clarify its diagnostic and prognostic significance.
